# Tele-triaging: a qualitative study exploring pharmacists’ clinical decision-making in a Poisons Information Centre using interviews and a clinical vignette

**DOI:** 10.1007/s11096-025-02000-3

**Published:** 2025-09-20

**Authors:** Qi Xuan Koh, Sarah Wise, Deborah Debono, Darren M. Roberts, Jane E. Carland

**Affiliations:** 1 Department of Clinical Pharmacology and Toxicology, St Vincent’s Hospital, Sydney, Australia; 2 School of Clinical Medicine, St Vincent’s Healthcare Clinical Campus, Faculty of Medicine and Health, UNSW, Sydney, Australia; 3 School of Public Health, Faculty of Health, University of Technology, Sydney, Australia; 4 New South Wales Poisons Information Centre, Sydney Children’s Hospitals Network, Westmead, Australia; 5 Edith Collins Centre, Drug Health Services, Royal Prince Alfred Hospital, Camperdown, Australia

**Keywords:** Decision making, Pharmacists, Poisons information centre, Qualitative

## Abstract

**Introduction:**

Specialists in Poisons Information (SPIs), most of whom are pharmacists, work in Australian Poisons Information Centres (PICs) and provide telephone-based triage (tele-triage) and management advice for poison exposures. Australian PICs answer calls from the public and healthcare practitioners and are considered an emergency telephone service. While tele-triaging and clinical decision-making have been explored in other health professions, limited studies are available exploring how pharmacists apply their knowledge to make clinical decisions in a busy tele-triage emergency setting.

**Aim:**

To explore how SPIs apply clinical judgement in tele-triage and to understand the factors that shape their decision-making.

**Method:**

This study represents the second, qualitative phase of an exploratory sequential mixed-methods design examining calls related to unintentional poisoning exposures in older adults (≥ 75 years) to the New South Wales PIC. Semi-structured interviews with 12 SPIs were conducted, supported by clinical vignettes and analysed using an inductive approach. Thematic analysis was combined with process mapping to describe the decision-making process.

**Results:**

SPIs followed a flexible, three-phase process of information gathering, risk stratification, and management decision-making. This process was iterative, shaped by experience, clinical knowledge, and the urgency of the call. Decision-making relied on the ability to balance known and uncertain risks, interpret caller information, and assess social and clinical context. While structured guidelines supported consistency, SPIs emphasised the importance of clinical autonomy, particularly in complex cases. A strong collegial culture and peer learning were central to developing decision-making skills. Time pressure and documentation requirements created tensions, highlighting the need to align workflows with clinical priorities.

**Conclusion:**

SPI decision-making is a dynamic, context-dependent process that combines clinical expertise, guideline use, and real-time judgement. Findings have implications for SPI training, documentation systems, and the design of telehealth services involving complex risk assessment.

**Supplementary Information:**

The online version contains supplementary material available at 10.1007/s11096-025-02000-3.

## Impact statements

- This study highlights the pivotal role of pharmacists at the NSW Poisons Information Centre (PIC) in tele-triage and provision of management advice for poisoning exposures in a time critical environment.
- SPIs’ clinical decision-making is flexible and evolves with experience. Unlike algorithm-driven systems, SPIs rely on nuanced clinical judgement, enabling tailored responses to diverse poisoning cases in real-time.
- The findings highlight the importance of peer-led training and collaborative workplaces, while revealing the need for continued improvement in systems to support clinical priorities and alongside administrative requirements.

## Introduction

Poisonings involve exposure to pharmaceutical products, chemicals, bites and stings, or other toxic hazards that may lead to harmful outcomes [1]. Poison Information Centres (PICs) are specialised emergency telephone advice services staffed by Specialists in Poisons Information (SPIs). SPIs perform tele-triage, manage poisoning cases, provide drug and poison information [2], and reduce healthcare costs by preventing unnecessary Emergency Department (ED) visits and ambulance use [1, 3]. PICs improve access to clinical advice for vulnerable groups, including low-income, rural, and older adult populations [4]. In Australia, PICs are estimated to save the healthcare system AUD $10 million annually (approximately EUR$5.5 million) [1].

Internationally, SPIs working in PICs have backgrounds from varied disciplines, predominantly in healthcare (e.g. pharmacy, nursing or medicine) and also science [5]. In Australia, SPIs are predominantly clinical pharmacists who undergo specialist training in toxicology [5]. They provide management and disposition advice to callers to the PIC and have access to additional support of clinical toxicologists as required [6]. SPIs use triage principles to assess the urgency of poison exposures and advise callers on whether the situation can be safely monitored at home or requires medical attention [1, 7–9].

Triaging is a context-dependent reasoning process that draws on clinical experience, guidelines, and existing knowledge to determine urgency and appropriate action [10–13]. Triage is typically performed face-to-face in emergency settings to prioritise care based on urgency [14, 15] but is now increasingly delivered by telephone (tele-triage) and facilitated by Artificial Intelligence (AI) [16]. Studies of nurse-led tele-triaging services like NHS Direct (UK) and Healthdirect (Australia) highlight the complexity of remote decision-making, even when supported by computerised algorithms [14, 17–20]. Likewise, emerging studies demonstrated the ability of AI to support medical triage in telemedicine [21] and to diagnose poisonings [16, 22], however its real-world applications to support tele-triaging in PICs have yet to be established.

### Aim

While previous research on pharmacists’ clinical decision-making has explored their role in therapeutic decision-making, and in the diagnosis and management of minor ailments [23], little is known about how pharmacists apply their clinical skills in the PIC environment [9]. To our knowledge no studies have specifically examined how pharmacists use their knowledge and clinical expertise to perform tele-triaging and provide management advice for an emergency telephone line. This study addresses this gap and responds to broader calls for a deeper understanding of pharmacists’ clinical decision-making [24] by exploring the decision-making processes of SPIs working in an Australian PIC.

## Method

This paper presents the second phase of an exploratory, sequential mixed-methods study on unintentional poisoning exposures in adults aged ≥ 75 years. Phase one, a retrospective audit of call records, identified call patterns, poisoning outcomes, and risk factors linked to hospital referral [25]. Findings in Phase One included that patients with similar poison exposures were provided with different advice by SPIs, and that over half of hospital referrals had a Poison Severity Score of ‘0’ (indicating ‘no effect’) at the time of the call. These suggest that in addition to the categorical factors examined in Phase One, other factors could influence disposition decision-making. Phase two built on these findings and used semi-structured interviews and a clinical vignette. Clinical vignettes are incomplete reports of a patient’s presentation aimed to reflect real-life situations to encourage discussions to problems with multiple possible solutions [26]. The interviews were aimed at exploring how SPIs apply clinical decision-making in tele-triage—assessing poisoning risk and advising on management and disposition.

### Study setting

The study was set in the New South Wales (NSW) PIC, the largest of four Australian PICs. The NSW PIC manages nearly 120,000 calls, which is approximately 50% of the calls received nationally each year.

The NSW PIC handles calls related to all types of poisonings, with callers including the affected individuals (the patient) or others calling on their behalf (e.g. family members, nursing home staff, paramedics, hospital personnel). Calls are answered by SPIs, most of whom hold degrees in pharmacy and are registered health practitioners. The PIC staffing varies between years but includes approximately 30 SPIs, 30 doctors and 2 administration officers. Many staff are part time or casual employees. There are other honorary clinical and research staff who make ad hoc contributions. All SPIs undergo additional on-the-job specialist training in poisons information and clinical toxicology.

### Interview guide development

The research team, comprising an undergraduate medical student (QXK), three health services researchers (JEC, SW, DD), and a senior clinical toxicologist (DMR), developed a semi-structured interview guide (Supplementary Material 1). The guide explored SPIs' perspectives on key results from the earlier audit [25], aiming to confirm, refute, or contextualise them and examine decision-making processes accessible through qualitative inquiry.

Interviews focused on challenges SPIs encountered when managing calls about older adults, the information used to guide decisions, and the role of formal guidelines. A clinical vignette was embedded in the interview. The vignette was based on a real case from Phase One of our research [25], including partial details such as age, location, poisoning intent, and substances involved (Supplementary Material 1). The think-aloud method [27, 28] encouraged participants to articulate their reasoning, assess whether the information provided was sufficient, and identify any additional details needed to guide their decision-making. No time limit was given to participant responses to the vignette or any part of the interview.

This vignette was reflected a common case reported to the NSW PIC in which the SPI had to decide whether the patient can be safely monitored at the site of call (a residential aged care facility) or if they need to be referred to hospital. We observed cases of varied decision-making between SPIs despite apparently similar exposure details and no symptoms or signs at the time of the call in Phase One [25]. Features that were common to such calls were cases involving oral ingestion due to a therapeutic error of cardiovascular drugs and did not involve other “high-risk” medications. However, we also selected other factors for inclusion in the vignette that were associated with increased risk of hospital referral in our research [25], such as exposure to beta blockers, as well as factors that were inconsistently recorded in the database such as the patient’s past medical history and the use of dose administration aids.

### Data collection

All SPIs at the NSW PIC (N = 31) were invited to participate via email, which included an information sheet, circulated via a senior SPI unrelated to the research. Interested individuals contacted QXK with questions before providing written consent. QXK did not share participant identity with any other members of the research team, ensuring SPI anonymity. Participation was voluntary, with no reimbursement. Participants could withdraw at any time.

Interviews were conducted by QXK between September and October 2022, either online (Microsoft Teams or Zoom) or in-person. Each interview lasted 30 to 80 min (mean: 50 min), was audio-recorded, transcribed, and de-identified using participant codes (e.g., SPI01). Recruitment continued until the sample included SPIs with varied experience and data saturation was reached, indicated by repeated insights [29]. Data collection and analysis occurred concurrently, with regular team meetings to refine codes and themes and determine thematic saturation.

### Data analysis

Due to the lack of prior research on pharmacists’ decision-making in tele-triage, a general inductive approach was used. Transcripts were de-identified and analysed in NVivo v12 following Braun and Clarke’s framework [30]. The Braun and Clarke framework of thematic analysis involved six steps of data familiarization, initial code generation, searching for themes, theme reviewing, defining and theme naming and finally report production. One researcher (QXK) transcribed and undertook preliminary coding of the data, with regular team discussions (SW, JEC, DMR, DD) to address challenges and review emerging findings to determine codes. Codes were grouped into categories, then into broader themes and subthemes, which were discussed and revised until agreed upon by all members of the research team.

To support understanding of SPIs’ workflows, QXK and SW created a process map during analysis to visualise how information is gathered and synthesised during calls [31]. The draft map was refined with the research team and validated at a senior PIC staff meeting, ensuring it accurately represented the call-taking process at NSW PIC.

### Reflexivity

All authors contributed to data analysis, offering varied interpretive perspectives. QXK, a fourth-year undergraduate medical student at the time, brought an ‘outsider’ view to tele-triaging and the SPI role, which helped clarify assumed knowledge. However, her lack of insider experience may have led to missed nuances, so transcripts were reviewed by the full research team, including those familiar with health services research and PIC practices. SW, JEC and DD and healthcare researchers unrelated to the PIC and SPIs so they offer ‘outsider’ views to processes. DMR is a senior employee at NSW PIC and a previous SPI, offering insights based on experience with PIC processes, and the impact of training and experience on SPIs’ decision-making. This allowed the team to have some knowledge of SPI and PIC functions and processes, with sufficient external reflection by appropriate experts.

### Ethics approval

The study was approved by the St Vincent’s Hospital Human Research Ethics Committee (2022/ETH01424), obtained on 28 July 2022 with written consent obtained from all participants.

## Results

Table 1 profiles the 12 SPIs interviewed, ten of whom were qualified pharmacists. Two SPIs were pharmacy graduates. The results are presented in two parts: first, a process map of SPIs’ call-taking; second, three themes from the interviews that offer insight into how SPIs apply and develop their clinical decision-making skills.

**Table 1:** Interview participant characteristics

Participant characteristics	Number of participants (N = 12)
Years working in the PIC	
Less than 1 year	2
1–5 years	4
6–10 years	3
More than 10 years	3
Experience prior to working in the PIC	
Community pharmacy	3
Hospital pharmacy	3
Community and hospital pharmacy	4
Others (pathology, research)	2

PIC, Poisons Information Centre

### Call-taking process map

**Fig. 1:**
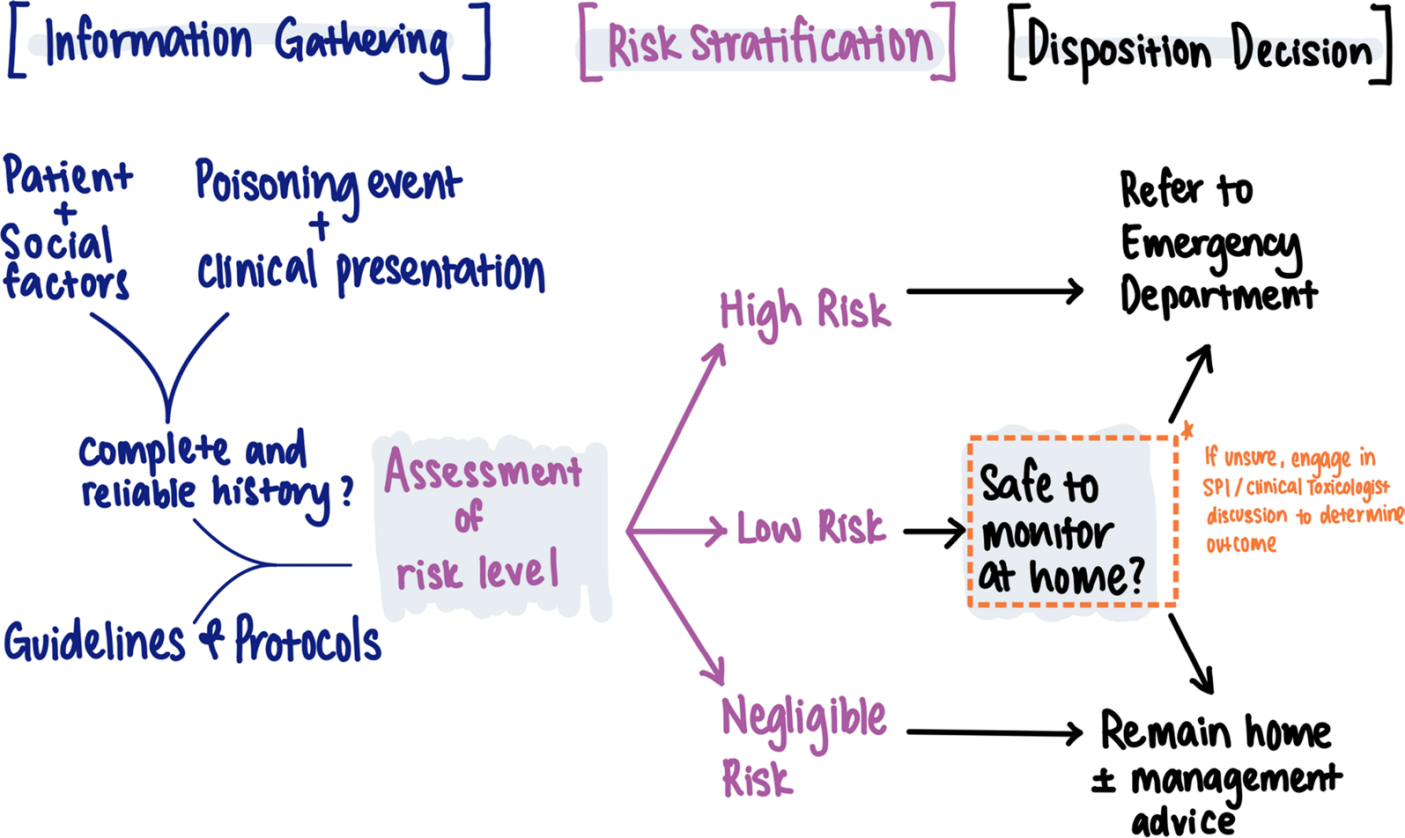
Schematic map of the decision-making process used by the Specialists in Poisons information in the New South Wales Poisons Information centre

Figure 1 illustrates the SPI call-taking process. Across experience levels, SPIs described an iterative three-phase process: information gathering, risk stratification, and management decision (Supplementary Table 2). The three phases were iterative rather than linear, with SPIs moving between them fluidly during a call.

In the information-gathering phase, SPIs aimed to understand “*what's actually happened here*” (SPI07). They used resources such as the Australian electronic Therapeutic Guidelines, Australian Medicines Handbook, Australian National Poisons Register, MicroMedex, Monthly Index of Medical Specialities (MIMS), and Toxinz™ to assess whether the poisoning met *“criteria for referral”* (SPI08).

SPIs asked a mix of open and specific questions to document mandatory case note fields, such as exposure type, timing, and substance (see Supplementary Table 1). SPIs also explored social and contextual factors relevant to risk, including time of day, distance from hospital, support availability, and access to tools such as a blood pressure monitor. These factors were recorded in the open-text section of the case notes, though completeness varied.

SPIs synthesised this information with clinical experience to assess poisoning risk, working independently without decision support software. When the risk was clear, they advised home management (lower risk cases) or referred to ED (higher risk cases). For uncertain or more complex cases, they consulted colleagues or escalated to a clinical toxicologist. In some cases, they arranged follow-up calls to reassess and adjust the plan.

Although Fig. 1 presents the call-taking process in a linear format for clarity, interviews revealed that SPIs apply this process flexibly rather than following a rigid framework. They adapt their clinical decision-making in real time, tailoring their approach to each patient and situation. The following three themes, drawn from the interview data, offer deeper insight into how SPIs develop and apply these skills: balancing known and uncertain risks, maintaining decision-making flexibility under pressure, and the influence of experience and work environment.

### Balancing known and uncertain risks

SPIs’ decision-making centred on triaging poisonings based on risk assessment. This involved evaluating both known risks of the exposure and potential uncertain risks that could lead to deterioration without medical care.

During information gathering (Fig. 1), SPIs assessed the patient’s clinical status and checked whether a toxic dose had been exceeded, using guidelines. The presence of *“red flag”* substances, such as opioids, insulin, or sedatives, prompted a lower threshold for hospital referral. However, SPIs stressed that identifying known risks alone was not enough to guide management. As one SPI explained:

“it’s not like you can just look at a textbook and say OK, this is the referral dose. If they’re under, leave them at home, if they’re over send them in…there’s a nuance” SPI06

SPIs described this “*nuance*” as balancing known risks with the uncertain risk of deterioration. Poisonings in older adults often required a more “*conservative*” approach (SPI03) due to their vulnerability to adverse effects. Confidence in the patient’s ability to self-monitor influenced decisions, with social factors explaining why similar exposures could lead to different advice. Key considerations included time of day, location, available support, ability to monitor at home, and access to care, as one SPI noted:

“…realistically in an aged care setting, there’s very limited staff overnight, there's a good chance there's not a registered nurse on duty. So, in reality, who's going to be monitoring him?” SPI05

SPIs emphasised that their confidence in the reliability and completeness of the caller’s information was just as important, if not more so, than the nature of the poisoning. They considered factors such as the caller’s anxiety and English proficiency, which could affect understanding. SPIs assessed consistency by summarising and clarifying the sequence of events and later evaluated whether patients had “*taken everything we’ve said on board*” (SPI12) when giving advice.

These factors influenced how confident SPIs felt about a patient's ability to be safely monitored at home. If there was any doubt, they often chose to “*err on the side of caution*” (SPI03) and referred the patient to the ED.

### Decision-making flexibility under pressure

SPIs described the challenge of balancing known and uncertain risks in a “*high-stakes, high-responsibility, high-speed environment*” (SPI11). Making risk assessments under time pressure required flexibility, a skill that developed with experience working as a SPI. This need for adaptability was often reflected in the brevity of call documentation, a trade-off necessary to manage high call volumes.

The stress of tele-triaging was heightened during peak periods, particularly for complex cases such as calls involving older adults. One SPI recalled the pressure they felt as a newcomer:

“…seven [people] in the queue and you’re the only person there…there’s a tendency to want to wrap up the calls and move on to the next one quite quickly” SPI07

While NSW PIC SPIs no longer work solo, these same pressures still exist when multiple callers are in the queue during busy periods. Administrative tasks further intensified time pressures. Documenting call details and completing mandatory fields, such as the Poison Severity Score, were required before logging each call:

“it’s not just taking the call, and that’s it…We have to type up the call record…type in all the fields…everything they said and plus your advice…and then you have to code it as well” SPI04

SPIs described the conflict between gathering sufficient information for the current call and completing documentation, while knowing that any delay could impact patients with more severe poisonings waiting in the queue. However, they noted that this tension eased with experience. Newer SPIs tended to follow a structured, linear approach to information gathering (Fig. 1) and frequently referred to guidelines. Over time, experience translated into greater flexibility in call-taking. SPIs adapted their framework, relied more on clinical judgment, and developed an intuitive sense of “*just knowing the questions to ask*” (SPI05) and distinguishing between “*need to know*” and “*want to know*” (SPI02) information.

Decision-making became increasingly instinctive, with experienced SPIs describing a “*hunch*” (SPI03) or a “*feel for what might be a risky situation and what might not…*” (SPI10). With this, the flow and efficiency of calls improved, and SPIs recognised when to resist the urge to rush the call, even under high call volumes:

“If it was a simple call, I would speed it up and move through it. But if I was talking to someone confused or hard of hearing, it wouldn’t make any difference to how long I spent on that one…try and focus on the person you’ve got…even if they [other calls] have to wait, a person still needs your help.” SPI07.

The flexibility afforded to SPIs, allowing them to triage patients autonomously rather than relying on computerised decision-support algorithms, enabled more individualised risk assessments. However, balancing patient care with operational demands often led to retrospective documentation, particularly during busy shifts. One SPI illustrated this trade-off:

“on a busy shift, like I worked one the other night I was actually pretty good, I had at about 12 or 14 incompletes. One of the girls I was working with, got to a point where she had 30 calls that she had to type up…but we had been so busy….she's experienced and she'd been pumping through them…so then going back like many hours later, and looking at the brief notes you’ve typed in there and trying to remember the details and document all of that… that's part of the reason that documentation doesn't really encompass everything that we would like to.” SPI01

SPIs were also asked to record information that did not always directly influence clinical decisions and could be added later. For example, while the Phase One audit found that over half of older adults referred to hospital had a Poison Severity Score of ‘No Effect’ [25], SPIs clarified that a low score simply meant symptoms had not yet developed and did not rule out the need for intervention.

“It’s a coding thing that we put in the database, but it doesn't guide our decision” SPI04

In the context of a Poison Severity Score of ‘No Effect’ in the vignette, they acknowledged the Score’s value for research and tracking trends but emphasised that, in practice, decisions were guided more by the uncertain risks and the “*expected trajectory*” (SPI02) of the poisoning, particularly in complex cases involving older adults.

### Decision-making experience and environment

Our findings highlight the critical role of experience and the work environment in shaping SPIs’ clinical decision-making. SPIs often “*draw from… experience, what’s worked in the past*” (SPI06) to decide when to conclude a call or spend more time assessing risk. While prior clinical experience as a pharmacist provided a foundation, structured training, peer review, and a supportive team environment were essential for developing tele-triage skills.

Previous pharmacy experience helped SPIs interpret key information, such as medication types, dosing, and packaging, and recognise signs of deterioration—balancing known and unknown poisoning risks. Those with hospital pharmacy experience noted that clinical exposure improved their ability to detect symptoms indicative of patient deterioration. Prior experience in interpersonal skills also helped assess whether callers were “*switched on*” (SPI05) or “*muddled and confused*” (SPI01), which could signal that “*there’s something else going on*” (SPI08). However, triaging acute, fast-changing cases was new for many and addressed through PIC training.

Most SPIs described the transition as a “*steep learning curve*” (SPI04), crediting peer support and team learning as crucial. Colleagues’ expertise was especially important when dealing with uncertainty in risk assessment, and shared case discussions helped build trust and foster system-wide learning. Peer learning was particularly valuable for newer SPIs, who felt “*privileged*” (SPI11) to learn from more experienced colleagues. Formal peer review (where each call record is checked by another SPI) ensured advice accuracy and supported skill development by helping new staff “*understand the thought processes*” (SPI06). Informal mentoring was also key, with senior SPIs offering feedback and role-playing calls to build confidence.

Experienced SPIs noted that learning was mutual, as newer colleagues contributed fresh perspectives:

“They’ve got their own clinical background that they sort of bring … I often learn a lot of stuff from new SPIs as well. So I think it goes both ways…” SPI06

## Discussion

With the pharmacy profession increasingly shifting toward roles involving clinical decision-making [24], this study describes an established tele-triaging and advice service in which pharmacists are supported to make rapid decisions on potentially high-acuity cases. Australian PICs are a unique environment where pharmacists, trained in toxicology, supported by clinical toxicologists, assess poisoning severity and provide management advice to both the public and healthcare professionals. This study identified that pharmacists synthesise their knowledge, clinical experience and guidelines to autonomously assess risk and make disposition decisions.

We identified a flexible, iterative three-phase model of information gathering, risk stratification, and decision-making. Although this structure aligns with cyclical decision-making models such as Wright et al.’s [24], our talk aloud method revealed that SPIs moved fluidly between phases, revisiting and refining decisions as more information emerged. Effective decision-making relied on clinical judgement shaped by training, experience, and peer learning, and was influenced by the time-sensitive nature of emergency calls. As SPIs gained experience, the three-phase call-taking model became more intuitive, allowing SPIs to transition seamlessly between stages and develop the clinical judgment needed to supplement and adapt formal decision-making guidelines. This is seen in other clinical contexts, with pharmacists’ experience aligned with increased efficiency and confidence roles [32, 33]. Like triage nurses, SPIs developed judgement through repeated exposure to similar cases, allowing them to prioritise key details, use intuitive (System 1) thinking for routine cases, and apply analytical (System 2) thinking for complex scenarios [15, 34–37]. Benner [38] describes this as an acquired ability to focus on clinically relevant elements, rather than treating all information equally.

The collegial culture at NSW PIC played a key role in skill development. Formal mechanisms such as supervised calls, peer review, and real-time consultation supported learning, while informal mentoring and role-play helped junior staff build confidence [10, 39, 40]. Junior SPIs valued mentorship, while senior SPIs also reported learning from new colleagues, highlighting a reciprocal learning culture [7, 41]. This collaborative approach contrasts with typical call-centre models, which tend to be more isolated and less supportive [42, 43]. Effective communication was central to SPIs’ work and reflected best-practice telehealth techniques identified in those previous studies, including the use of open- and closed-ended questions, summarisation, teach-back methods, and attention to auditory cues reflected [8, 11, 44, 45]. With experience, SPIs refined their listening and questioning techniques, improving their ability to detect subtle signs of poisoning severity or risk.

Unlike algorithm-driven services such as NHS Direct [46–48], SPIs predominantly rely on their clinical judgement, with guidelines supplementing decision-making. Although AI decision-support tools boast the advantage of wider exposure to clinical scenarios that an individual can encounter [22], its decisions may lack nuance due to failure to consider factors beyond the poisoning event and current clinical presentation [16, 22]. Thereby, not accounting for the unknown risks of deterioration, including the accuracy of information provided, the patient’s social factors, as well as the patient’s ability to monitor for symptoms of deterioration. Moreover, while decision-support tools offer consistency [47, 49, 50], over-reliance can lead to dissatisfaction, deskilling, and reduced responsiveness to case nuances [15, 19, 47, 51]. Research suggests that call-takers in tele-triage and advice services may selectively apply algorithms that align with their own clinical reasoning [15, 20, 52–54], and that protocol use does not always improve patient outcomes [48, 55]. In our study, SPIs valued guidelines but noted they do not fully capture poisoning complexity, particularly in grey-area cases. Clinical autonomy remains essential, especially for junior SPIs developing their judgement [44].

Our findings also offer insight into the challenge of balancing tele-triaging with mandatory documentation under time pressure. Timely documentation of events as they occur in real-time ensures an accurate reflection of the patient’s condition and care delivered [56]. This is crucial for clinical communication between healthcare workers, medico-legal and reimbursement purposes [57], and facilitates identification of improvement opportunities [58]. However, Soucek et al. [59] demonstrated that time pressure and staffing levels negatively impact documentation behaviours such as retrospective documentation which may result in difficulties reconstructing events later. The misalignment between required data fields and SPIs’ clinical priorities for immediate decision-making suggests a need to better align documentation processes with decision-making workflows, as well the adequacy of staffing levels during peak call times. This could in turn, contribute to a reduction in administrative burden experienced and the time and effort involved in clinical documentation [59, 60]. For example, although the Poison Severity Score is useful for population-level comparisons [61], it does not necessarily guide real-time management, in particular for low scores. SPIs focus instead on contextual factors, such as poisoning trajectory and patient vulnerability, often not captured in mandatory, structured data fields. As SPIs provide immediate risk assessments rather than long-term monitoring, documentation should prioritise decision-critical information. The updated NSW PIC database allows SPIs to save draft reports and move to the next call (personal communication), which may help reduce this burden. Future research should evaluate how documentation tools support clinical workflows and call efficiency.

### Strengths and limitations

The use of a mixed-methods approach helped to contextualise the findings from Phase One of the study. Moreover, given the limited work available on pharmacists’ decision-making in poison centres, the use of a vignette and the think-aloud approach helped to provide further insight into this matter.

Interviews conducted in 2022 may not fully reflect current SPI experiences, but we have no reason to think they would have changed and the process map findings were validated in a 2024 executive meeting. As the study was conducted at a single Australian PIC, findings may not be immediately generalisable to other poison centres. Thus, efforts were made to capture perspectives from SPIs across varying experience levels to enhance the transferability of findings and interviews were conducted with nearly half of the SPI workforce. Additionally, a single vignette was used in this study, which may limit the generalisability of findings given the context-specific nature of decision-making. However, this was done to regulate the duration of interviews and balance respondent participation. Moreover, the vignette was used not to capture and compare the pharmacists’ decision, rather to stimulate discussion of case characteristics and factors that influenced the decision-making process. Thus, the discussion of the vignette increased the generalisability of findings to other scenarios. Coding was led by a single researcher (QXK). However, regular team meetings were conducted to discuss the codes as they were developed, allowing for discussion of coding challenges, re-coding and co-development of emerging themes. Notably, the draft call-taking process map (Fig. 1) was generated by two researchers (QXK, SW) before being brought to the wider research team. Future studies in this area could consider the use of multiple case vignettes to increase representation of calls received by the NSW PIC, as well as identifying discrepancies in theoretical decision-making and actual practice.

## Conclusion

The NSW PIC offers a unique context for examining how pharmacists apply clinical decision-making in tele-triage. Our findings reveal a dynamic process in which SPIs balance known and uncertain risks, adapt their approach based on experience, and provide urgent medical advice to often underserved populations, helping to prevent unnecessary hospitalisations. The interaction between structured guidelines and clinical judgement highlights the distinctive nature of SPI decision-making, setting it apart from algorithm-driven tele-triage services. Streamlining documentation to prioritise clinically relevant information may improve efficiency, reduce administrative burden, and strengthen the integration of qualitative and quantitative data.

## Supplementary Information

Below is the link to the electronic supplementary material.

Supplementary file1 (DOCX 30 kb)

## Data Availability

Due to the nature of the research and ethical restrictions, supporting data is not available.
