# Supplementary material for: Tele-triaging: a qualitative study exploring pharmacists’ clinical decision-making in a Poisons Information Centre using interviews and a clinical vignette
Source: Int J Clin Pharm. 2025 Sep 20;48(2):469–78. doi: 10.1007/s11096-025-02000-3 (PMC12992380; doi:10.1007/s11096-025-02000-3)
Supplement: Supplementary file 1 — Supplementary file1 (DOCX 30 kb) [file 11096_2025_2000_MOESM1_ESM.docx]

## Supplementary Table 1 NSW PIC mandatory field data

| **Categories** | **Mandatory data fields** |
| --- | --- |
| Call characteristics | Date of call |
|  | Time of call (nearest minute) |
|  | Call type (Exposure or recalls) |
|  | Patient’s state in Australia |
|  | Caller identity (e.g. family, friend) |
| Patient demographics | Sex |
|  | Age |
| Poisoning characteristics | Poisoning type  (therapeutic error, accidental poisoning, adverse drug reaction) |
|  | Dose of substance |
|  | Substance name and active ingredient |
|  | Time since exposure |
|  | Administration route |
|  | Does patient present symptomatically |
|  | Management recommendation |
|  | Poison severity score |
| Free text descriptions | Notes taken by Specialists in Poisons Information (SPI) during the call  (e.g., Location of call, use of dose administration aids, social circumstance) |
|  |  |
|  |  |

## Supplementary Table 2 Description of phases in the decision-making process, information considered and supporting quotes by SPIs (n=12)

| **Information Gathering Phase** | | |
| --- | --- | --- |
| **Poisoning event** | What drug(s)? | “What’s actually happened here? Are these medications that belong to another resident that had been given in error? Or have they received double doses of the, their own medications? Have they all been given at once?” SPI07    “There are certain meds that are definitely like oh, you have to send them straight to hospital.” SPI04 |
|  | What dose(s)? |  |
|  | Who was the drug prescribed for? |  |
|  | How long has patient been on these drugs? |  |
| **Clinical presentation** | Age | “When we talk about older adults, you know when they're 75, 80 that's when we worry. Because with any medication, they're more susceptible.” SPI03 |
|  | Patient’s comorbidities | “Their various comorbidities… puts them at higher risk of an adverse event...” SPI02 |
|  | What are the patient’s current symptoms? | “And how he is at the moment [short pause], does he have any sort of new symptoms or changes in his condition currently?” SPI05 |
|  | What potential symptoms can occur? | “What their expected trajectory is, it’s probably almost more important.” SPI02 |
| **Social factors** | Time of day? | “We’ll have a lower threshold for referring them to… the hospital…if they’re home alone, especially if it’s nighttime.” SPI02 |
|  | Distance from hospital? | “If they’re further, we’re more like to send them. Because if someone lives 5 minutes from hospital and something happens, they have health access quite quickly.” SPI08 |
|  | Staffing levels at nursing home? | “I get scared that there aren’t enough nurses in residential aged care facilities to monitor patients effectively.” SPI07 |
|  | Level of support? | “Most of the time you can keep someone at home if there's someone at home to keep an eye on them, if they've got a blood pressure machine, if they're close to hospital.” SPI11 |
|  | Availability of monitoring tools  (blood pressure monitor etc.) | “If we got an elderly adult who might be immobile or might have some cognitive impairment or whatever and we’re concerned that there may not be the ability to have the level of monitoring that we require.” SPI01  “If I’m not confident that the monitoring is not in place and that they could potentially miss something and end up becoming very unwell at home.” SPI12 |
| **Reliability of information** | Can the information collected from the patient be trusted?  Influenced by comprehensibility and cognitive impairment | “The person was just too muddled and confused to be able to give you any clear answer. And in those situations, I’ve just called the ambulance and say, “can you go and check on this person.” SPI07    “Confusion. Has that led to their double dose, do they need to go to hospital because there’s something else going on?” SPI08  “It's making sure that the extra layers are there in place. So how close they are to hospital? Who's at home with them? Are we confident that they know what we're talking about with monitoring? Have they taken everything we've said on board?” SPI12 |
| **Guidelines and protocols** | Internal protocols in the form of protocol cards | “And you know so we do have some protocol cards where, you know, if we hear like a certain medication, we have to make a risk assessment on that.” SPI04 |
|  | External guidelines including the:  Electronic Therapeutic Guidelines, Australian Medicines Handbook, Toxinz,  Monthly Index of Medical Specialties  Australian National Poisons Register | “We have the Therapeutic Guidelines online so we have access …Australian Medicines Handbook (AMH), Monthly Index of Medical Specialties (MIMS), Therapeutic Guidelines so that's pretty much our go to the Therapeutic Guidelines toxicology section.” SPI04 |
| **Risk Stratification Phase** | | |
| **High risk** | Patient requires hospital referral | “My logic is if they are unwell now or they're likely to be unwell, then if they're at home, I would send to hospital.” SPI09    “You have clear black-and-white situations with something very, very acutely toxic and you absolutely need to go to hospital and those are very, very clear.” SPI10 |
| **Low risk** | Patient may be able to watch and wait for symptom development | “You'll have those grey areas like a double dose or we're not sure if they're going to tolerate it or not…then yeah those subjective factors will come into it.” SPI10 |
| **Negligible risk** | Patient exposure is deemed non-toxic | “(when discussing about a call to patients with non-toxic exposure) It's not even toxic. You don't even have to worry. You guys can go home.” SPI11  “Actually this is going to be a well-tolerated ingestion, I'm not actually expecting anything to happen.” SPI10 |
| **Final Decision Phase** | | |
| **Hospital** | Hospital referral needs to be arranged | “It could be life-threatening, so you’d refer them to the hospital.” SPI05 |
| **At home/GP** | Patient can be monitored at home or should arrange follow-up with GP | “If I'm expecting that nothing's going to happen and it's going to be a relatively benign ingestion, then eventually they can stay at home with monitoring.” SPI10  “(with regards to when they would recommend GP referrals) Things that don't need an immediate medical assistance. But then you don't want someone to just sort it out themselves.” SPI11  “people have called us when they've actually explained more moderate to significant symptoms (before the call). And it's more that they called us in a delayed fashion that… they've obviously come through, they've tolerated a lot of the symptoms luckily. Now I think it's just appropriate to refer to the GP.” SPI12 |

*SPIs; Specialists in Poisons Information (n=12). GP; General practitioner*

## Supplementary Material 1 - Unintentional Poisonings in Older Adults - Interview Guide

Thank you for taking the time to talk with me today. This study is looking to gain insight from Specialists in Poisons Information (SPIs) about the trends we have observed when analysing calls relating to unintentional poisonings in older adults.

We are interested in understanding what it is like to receive these calls and the factors that help guide your decisions. And we will be doing so through questions as well as presenting a case scenario. We also noticed certain trends while analysing the data and wanted to gain your insight into it.

The interview will take about 30 – 45 minutes. Please be as open and as honest as you like. We are not collecting any identifiable information from you and your responses will not be linked to you in any way.

Do you mind if the interview is audio-recorded? With your permission, we will record the interviews because we don't want to miss anything you say. People often say very helpful things in these interviews, and we can't write fast enough to get them all down.

Do you have any questions before we begin? (If no, continue)

1. Can you tell me more your background as a pharmacist?

[Prompts: Now and in the past Community pharmacist? Hospital pharmacist? Clinical work? Complex medication regiment]

[Any post graduate qualification in clinical pharmacy]

1. How long have you worked at the NSW Poisons Information Centre?
2. What does the role involve?
3. What are some of the challenges you experience in taking calls from older adults?

[Prompts: Multiple drugs involved, unclear/incomplete information, polypharmacy, difficulty getting accurate information, different agenda from different callers]

- 1. How do you overcome these challenges?

[Prompts: Formal support – protocols, database; Informal support – seniors, colleagues]

1. Are there any differences managing these calls compared to other age groups?
   1. [Prompts: Is there any difference managing a call regarding children ≤5y.o. or adults 30-40 y.o.]
2. Do you feel like you have enough time per call?
   1. [Prompts: Have you felt rushed making decisions? Any noticeable patterns?]
3. Overall, do you feel confident when handling calls from older adults?
   1. What would make you feel more confident?

***Poison Centre Call Vignette***

The next section of the interview is not a test; rather it is designed to understand what information and factors help guide your decision-making when you receive calls.

What I am going to show you is the information provided during a call to the NSW Poisons Centre. [Proceed to show Vignette 1. Information to be shared will be non-identifiable data modelled on a call received by the NSW PIC during 2021]

**Case vignette (partial details)**

| **Vignette 1** | |
| --- | --- |
| **Call details** | **Call date:** Wednesday  **Call time:** Unknown  **Call made by:** Residential aged care facility nurse |
| **Patient details** | 91M  Resident in residential aged care |
| **Poisoning circumstances** | Therapeutic error |
| **Drug details** | Minax (Metoprolol) - 50mg x2  Spiractin (Spironolactone) - 25mg x1  Gabapentin 100mg x4  Allopurinol – 100mg x2  Lengout (Colchicine) - 500mcg x2  Doublut (Dutasteride + tamsulosin) x1  Euthroxsig (Thyroxine) 75mcg x2  Coloxyl and senna (x4)  **Route of administration:** Ingestion |

**Full vignette case (full case details, not shown to interview participants)**

| **Vignette 1** | |
| --- | --- |
| **Call details** | **Call date:** Wednesday  **Call time:** *Unknown*  **Call made by:** Residential aged care facility nurse |
| **Patient details** | 91M  Resident in RACF  *Comorbidities unknown* |
| **Poisoning circumstances** | Therapeutic error  *Given/taken multiple doses: Thursday and Friday slots in webster pack empty* |
| **Drug details** | **Total number of drugs:** 10  **Drug name and dose**  Minax (Metoprolol) - 50mg x2  Spiractin (Spironolactone) - 25mg x1  Gabapentin 100mg x4  Allopurinol – 100mg x2  Lengout (Colchicine) - 500mcg x2  Doublut (Dutasteride + tamsulosin) x1  Euthroxsig (Thyroxine) 75mcg x2  Coloxyl and senna (x4)  ***Unknown time since exposure***  **Route of administration:** Ingestion  ***Initial assessment:*** *Asymptomatic*  ***Poison severity score:*** *None (0)* |
| **Management outcomes** | *Call after hours GP to check vitals* |

1. Is the information in the case above sufficient to make a management decision?
   1. Which key pieces of information are needed to guide your decision-making process?

[Prompt: Age, drug names, number of drugs ingested, number of doses, symptoms, route of administration, drug combination, comorbidity (hepatic/renal impairment)

- 1. Is this information usually available to you?
  2. What are some of your main considerations?
     1. Any red flag drugs/circumstances and how are these important?

1. What additional information would be of use?
   1. [Prompt: Cognitive impairment, use of webster packs, time of day, additional risk factors, hepatic/renal impairment]
   2. How useful is it to gather information on patient’s:
      1. Current medical history and drugs taken
      2. Webster packs
      3. Cognitive impairment

**Explaining trends**

1. What are some of the key factors that make you decide whether a patient needs to be referred to hospital rather than to monitor at home or wait to see a GP
2. How does the poison severity score inform your decision?
3. We have analysed 6 months of data from the NSWPIC and noticed that some patients were referred to hospital despite having “no effect” on the poison severity score. Why would that happen?

**NSWPIC and the broader health system**

1. In your opinion, what contribution does the poison centre make to the broader health system?
   1. How could it be improved? [Prompt: data collection process]
2. Is there anything else you would like to talk about or that you think is important when you consider calls you receive as a SPI from older adults?

Thank you again for participating.
